# From abandonment to adoption: advancing assistive technologies for blindness and low vision in the AI era

**DOI:** 10.3389/fdgth.2025.1719746

**Published:** 2026-01-12

**Authors:** Roni Barak Ventura, Giles Hamilton-Fletcher, John-Ross Rizzo

**Affiliations:** 1 School of Applied Engineering and Technology, New Jersey Institute of Technology, Newark, NJ, United States; 2 Department of Rehabilitation Medicine, NYU Grossman School of Medicine, New York, NY, United States; 3 Department of Mechanical and Aerospace Engineering, NYU Tandon School of Engineering, Brooklyn, NY, United States; 4 Center for Urban Science and Progress, NYU Tandon School of Engineering, Brooklyn, NY, United States; 5 Department of Biomedical Engineering, NYU Tandon School of Engineering, Brooklyn, NY, United States

**Keywords:** assistive technology, technology abandonment, technology adoption, user-centerd design, artificial intelligence, blindness and low vision

## Abstract

Assistive technologies can enhance safety, independence, and quality of life for people with blindness and low vision. Despite their benefits, abandonment of these technologies remains widespread, and recent research on this issue is limited. In this Perspective article, we draw on both professional experiences and relevant scientific literature to examine adoption and abandonment in the context of new artificial intelligence-powered applications. We highlight risks arising from misaligned design, inconsistent industry support, and inadequate user training. We synthesize existing knowledge on factors that influence abandonment and propose three priorities to realign assistive technology development: participatory and transdisciplinary research, integrated technology ecosystems, and socially supported engagement. Taken collectively, these priorities ensure that emerging assistive technologies better align with the needs of people with blindness and low vision, promoting lasting adoption rather than abandonment.

## Introduction

Assistive technologies (AT) offer significant benefits to people with blindness and low vision (pBLV), enhancing their safety, functionality, independence, and overall quality of life (1–4). However, adoption of AT has historically been limited and constrained by several design and acquisition factors. Even though ATs can provide tangible benefits to pBLV and assist them with activities of daily living, many devices have been abandoned, primarily owing to concerns surrounding design, usability, aesthetics, low involvement of users in device development or device selection, and inadequate training (1, 5, 6). Insurance providers often view abandoned devices as wasteful investments and may refuse to fund their future procurement (5, 7). Thus, insurers play an even greater role in determining access to and adoption of AT. At present, while a few (e.g., Department of Veterans Affairs) offer broad coverage, the majority of providers (e.g., Medicare) constrain allowable expenses to AT that support in-home living (8). Consequently, the leading source of AT funding across all disabilities is out-of-pocket spending by individuals or their families, with ∼40% of users reporting this as their primary funding source (9). Ergo, when devices are abandoned, pBLV face a dual loss. First they forfeit both the financial investment and the functional benefits the technology was meant to provide. Second, widespread abandonment generates an ecosystem loss, as it undermines confidence among users and clinicians, setting back the entire field of AT and delaying progress toward more effective, inclusive solutions.

Recent advances in artificial intelligence (AI), particularly in natural language processing and computer vision, have enabled a new generation of ATs that support pBLV in unprecedented ways. Popular smartphone applications such as Be My Eyes or Seeing AI now offer AI-based image descriptions with support for answering follow-up queries from the user. Alongside a host of other assistive modes focused on daily tasks such as identifying products and currency, scanning documents, and reading text, modern AI advancements have unquestionably put a dent in information inaccessibility for pBLV (10). Navigation and mobility assistance have also advanced, with methods for sonifying outdoor GPS routes and places of interest (e.g., Soundscape community), negotiating pedestrian signals (e.g, OKO) as well as wayfinding within indoor scenarios for supported environments (e.g., Goodmaps, WayMap, NaviLens).

Beyond applications, AI has accelerated the development cycle for new AT and the volume of novel mobile applications is overwhelming the market. However, the drive to release technologies rapidly has clearly outpaced the creation of thoughtful, user-centered solutions. For example, Meta's smart glasses, which has its own AI for image description and context, has also partnered with BLV-focused solutions such as Aira and Be My Eyes to enable hands-free guidance. Although the technology shows great promise in enhancing user confidence across a limited range of daily functional activities (11), the glasses require a stable internet connection to fully utilize AI-driven features and demonstrate difficulties in identifying complex or novel objects in varied lighting conditions, particularly during tasks that require dynamic movement. Reported issues have raised concerns about insufficient safety testing or refinement for users before release, undermining their potential adoption by pBLV.

With these publicly available applications and more versions in development [e.g., Chatmap (10), Point-to-Tell (12), UNav (13, 14)], AI-assistance is becoming ubiquitous, fundamentally changing the AT landscape and offering voluminous opportunities for adoption by pBLV. The rapid release of new AT has increased adoption by expanding choice and driving down prices, but it has also introduced greater risk whereby devices enter an aggressive market, fail to meet user needs, and become abandoned. As a result, the very forces that make AT more accessible can simultaneously accelerate its abandonment. Considering these new dynamics, there is an urgent need to now revisit factors that drive adoption and abandonment of ATs by pBLV, especially given their potential to enhance user autonomy, function, and overall quality of life.

## The state of knowledge on AT abandonment

Herein abandonment is considered non-use of a device, or its replacement with a qualitatively different device type (5, 15). Foundational literature on AT abandonment is both limited and outdated. Research from the 1980s and 1990s reported that overall AT abandonment rates were around 29% (5), with rates for optical AT for pBLV ranging from 17% to 50%. More recent evidence from 2018 suggested that among secondary AT (e.g., magnifiers and filters) that were provided by a low vision rehabilitation clinic, only 17% of devices were abandoned at three months (16). For an interesting comparison, when it comes to hearing aids, contemporary abandonment reaches alarming rates of up to 78% (17). Anecdotal evidence suggests that digital apps may be abandoned at similar (if not higher) rates than hearing aids (18, 19). However, the literature is particularly scarce when it comes to abandonment of digital AT or smartphone applications for pBLV. This is surprising given that 87% of pBLV are interested in using AI-assistants (20). Interviews and diaries have indicated concerns and barriers to effective use of digital tools and AI-assistants (4, 21). As the technical world has matured to deliver transformative and accessible AT, what is needed now is not simply more applications and products, but better data and hypothesis-driven studies to understand the underlying drivers of abandonment.

As many claims about AI-related abandonment are currently inferential, we emphasize that these concerns remain hypotheses that empirical research is needed to test directly. Longitudinal adoption studies that track users' engagement with AI-powered assistive tools over time, as well as analyses of usage logs from commercial or research platforms, could help determine the extent to which issues such as poor user experiences, rapid feature turnover, or the availability of alternative platforms contribute to abandonment. Such data-driven approaches would allow us to distinguish between assumptions about AI-related abandonment and establishing evidence-supported mechanisms. Studies of AI-enhanced AT will also likely need to consider wider barriers to adoption, including user concerns around anonymity, data privacy, reliability, appropriateness, and the ethics of AI-usage in society (22, 23).

**Figure 1:**
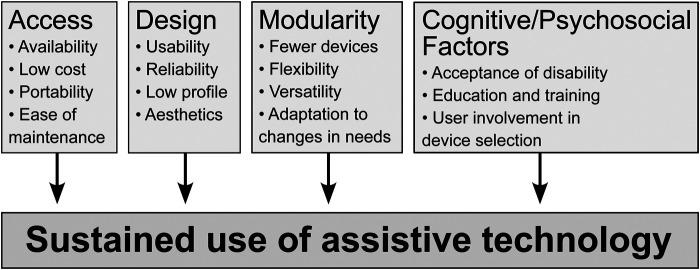
Factors identified in research as contributing to sustained use of assistive technology.

Research on AT abandonment has identified multiple factors that underlie technology abandonment (Figure 1), and a consistent theme across studies is that poor design, lack of flexibility, and inadequate usability contribute significantly to abandonment, as well as appearance and aesthetics. ATs that are not available, portable, affordable, or easy to maintain are at a greater risk of being discarded (1, 5, 24, 25). ATs that fail to adapt to changes in the lives and needs of users also face higher rates of abandonment (1, 5, 15, 26). These include changes in medical status or functional abilities (e.g., improvement or decline in sight condition), change of job, and/or change of insurance. ATs that demonstrate poor technical performance, are cumbersome to use, or can be replaced with simpler behavioral strategies (e.g., physical search), are typically abandoned (5, 6). Likewise, ATs that do not offer functional versatility and can only be used in a narrow range of environments or contexts often end up abandoned (27, 28). Finally, ATs that draw attention to the disability of pBLV and prevent them from being perceived as “normal” in public are less likely to be adopted in the long-run (29, 30).

In addition to practical design, research has demonstrated that cognitive and psychosocial factors play an important role in AT abandonment (31). Several studies have shown that user involvement in selecting AT, as well as the adequacy of training and follow-ups, are critical for sustained usage (1, 5, 15, 32, 33). Although individuals who are visually impaired are likely to embrace ATs, without proper education and training, they may struggle to effectively apply the AT in real-life scenarios. Many ATs, particularly mobile applications, are offered without any training, leaving users to navigate complex functions on their own. Similarly, newer market offerings such as Meta's devices also lack structured training support. As a result, not only does abandonment persist but it may also be rising at alarming rates, placing pressure on both users and the broader market. A recent study extended upon the Unified Theory of Acceptance and Use of Technology (UTAUT) model and found that both performance expectancy and training predict behavioral intention to use ATs (34). Other factors that are positively associated with sustained use of AT include acceptance of one's disability and proactive management of it (1, 15). By contrast, patients relying on multiple AT devices at once and perceiving limited value in each are both linked to higher risks of abandonment (35).

Of note, given the scope of this article, the views expressed here are drawn from our professional experiences and relevant scientific literature.

## Rethinking research priorities

Recent research has begun exploring how pBLV evaluate and engage with AI-powered AT (36–38), yet abandonment remains understudied. As new AT proliferates, it appears that the research landscape has drifted away from user-centered evaluation; devices are frequently designed based on technological capabilities rather than actual user needs, often resulting in misaligned solutions. This trend reflects a broader pattern in which innovation is driven by what is technically feasible rather than what is most useful or necessary for end users. Powerful companies with financial means and stake such as Microsoft and OpenAI have used AT for mass-market appeal as well as a testing ground for broader technological development. This has resulted in inconsistent support for AT tools: for example, Microsoft discontinued Soundscape while retaining Seeing AI, despite strong community support for both. Similarly, Toyota invested nearly $300 million in the BLAID project, a body-based visual AT solution that was designed to fill the gaps left by canes, dogs and basic GPS devices for pBLV, but ultimately canceled the initiative before its release. These hot-and-cold, push-and-pull market offerings squeeze smaller companies and offerings that may have been more viable and impactful to the priority population.

## Moving forward: design for sustained use

**Figure 2:**
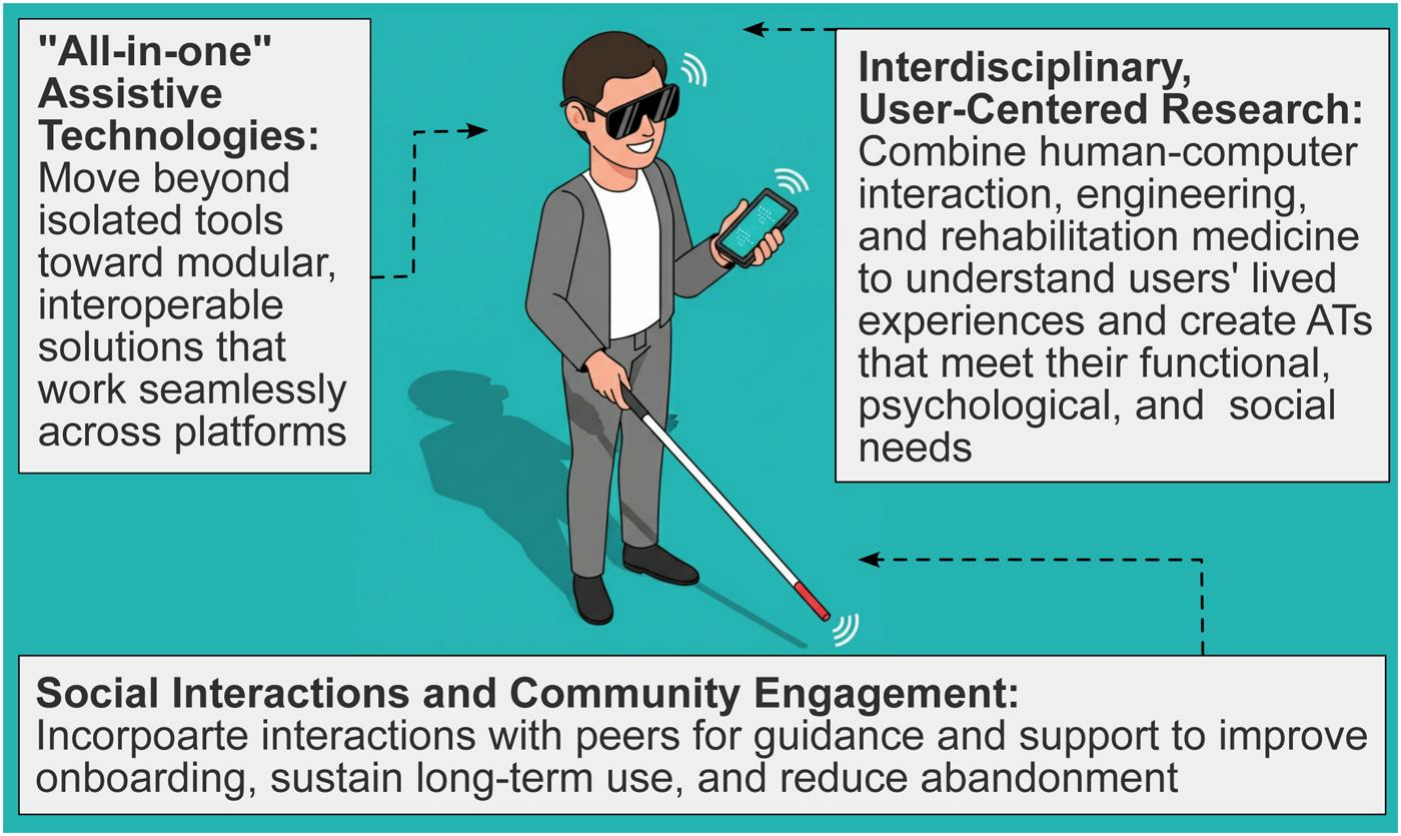
Three changes in research and development of AT that could reduce abandonment.

The hallmark of successful AT lies in its sustained, day-to-day use. To meaningfully improve the lives of individuals with visual impairment, we must realign AT research and development towards long-term engagement. We identify three necessary changes to achieve this goal, minimize AT abandonment and, ultimately, boost their impact and outcomes for pBLV (Figure 2).

First, applied research should integrate perspectives from human-computer interactions, engineering, and rehabilitation medicine. The research should involve participatory design coupled with real-time data about AT usage and arising issues, to provide a richer understanding of the end user-focused lived experiences (15). Such collaborative efforts could ensure that new AT solutions address not only functional needs, but also account for the psychological and social dimensions of use (31). Importantly, research should extend beyond measuring the effectiveness of AT and its impact on daily function, to also examine efficiency and the time required for users to become proficient with newly acquired technologies (39, 40).

Second, solutions must move beyond single-point tools toward integrated, multi-point AT ecosystems. That is, pBLV cannot reasonably adopt and manage dozens of separate applications or technologies in daily life. Instead, solutions must become modular and compatible across platforms so that ATs remain viable and sustainable over time. Research should therefore prioritize integration of AT hardware and software, as well as streamline user experiences to ensure AT works cohesively rather than in isolation, centering on interoperability. Building on this, research could lay the foundation for thorough pre-market testing, supported by evaluation frameworks that incorporate clear key performance indicators to guide both development and adoption. Examples of measurable indicators include short-, medium-, and long-term retention (e.g., 30-, 90-, and 365-day continued use), task performance metrics such as task-completion rate or time-to-completion, frequency and severity of errors or safety-related events, training duration, user satisfaction scores, and changes in quality-of-life or functional-independence indices.

Finally, AT should be designed to anticipate and mitigate abandonment by combining technology with social engagement. Specifically, AI could be leveraged to monitor usage patterns and respond to user needs by providing personalized support to sustain engagement. At the same time, social interactions have the potential to enhance user engagement and well-being. Studies with other populations with disabilities who experience high levels of isolation show that social support and peer mentoring can promote self-efficacy, adherence to routines, and improved rehabilitation outcomes (41, 42). Although the evidence specific to pBLV is limited, existing research suggests that group-based exercise can enhance adherence and that coaching can promote compliance with healthcare recommendations in this population (43, 44). Yet, the potential of such approaches has not been fully explored for AT. By integrating such social elements into training and on-boarding processes, users can learn new devices more effectively, receive peer support, and develop confidence in using the technology. In this manner, social AT could not only improve adoption and prevent abandonment, but also create a sense of belonging and community for those who experience exclusion, isolation, and loneliness (45, 46). For example, ATs that combine physical activity with social interactions within a social-fitness framework could serve dual roles in enhancing user health and well-being, while also reducing abandonment. As such, technologies that support activities that require long-term commitment (e.g., exercise apps) could offer a model for sustainable adoption of specific technologies.

## Conclusions

The success of AT hinges not only on innovation but also on sustained daily use. As AI-powered solutions become more prevalent and voluminous, it is essential to examine their unique benefits, drawbacks, and risks, including that of abandonment. Effective studies need to be grounded in transdisciplinary, user-centered research within a broader holistic framework that reflects the lived experience of AT usage. Such a framework must include training and education of users, evolving AT that adapts with user needs and environments, and a focus on harnessing social interactions and community engagement. Through this, we can identify AT barriers and potential facilitators that ultimately will catalyze the adoption and long-term maintenance of AT, improving autonomy, daily functioning, and quality of life of its users, and the sustainability of a technologically forward approach to vision care, staving off abandonment.

## Data Availability

The original contributions presented in the study are included in the article/supplementary material, further inquiries can be directed to the corresponding author/s.
